# Extreme Heat and Calls to Law Enforcement Related to Domestic Violence

**DOI:** 10.1001/jamanetworkopen.2025.30530

**Published:** 2025-08-29

**Authors:** Arnab K. Dey, Namratha Rao, Edwin Elizabeth Thomas, Yiqun Ma, Grace Riley, Tarik Benmarhnia, Anita Raj

**Affiliations:** 1 Scripps Institution of Oceanography, University of California, San Diego, La Jolla; 2 Newcomb Institute, Tulane University, New Orleans, Louisiana; 3 Department of Gender and Women’s Studies, University of Wisconsin–Madison; 4 Irset Institut de Recherche en Santé, Environnement et Travail, UMR-S 1085, Inserm, University of Rennes, EHESP, Rennes, France

## Abstract

**Importance:**

Although research has documented links between climate factors and violence, evidence specific to domestic violence (DV) remains limited, particularly in climate-vulnerable urban areas.

**Objective:**

To analyze the association between extreme heat events and DV-related calls to law enforcement in New Orleans, Louisiana, between 2011 and 2021.

**Design, Setting, and Participants:**

This cross-sectional study used a spatially weighted time-stratified case-crossover design analyzing DV-related call data between January 1, 2011, and December 31, 2021, from the New Orleans Police Department. Statistical analysis was conducted from March to May 2024.

**Exposures:**

Daily mean Universal Thermal Climate Index (UTCI) data were used to define 6 extreme heat definitions based on thresholds (30 °C and 90th percentile) and durations (1, 3, or 5 consecutive days). Additional thresholds were considered in sensitivity analyses.

**Main Outcomes and Measures:**

The primary outcome was DV-related calls made. Calls were aggregated to zip code days, and conditional logistic regression weighted by cases per zip code day was used to evaluate the association between extreme heat and DV calls. Attributable fractions and attributable numbers of DV calls due to extreme heat exposure were calculated.

**Results:**

Of the 150 523 DV-related calls during the study period, 69.6% were classified as domestic disturbances, and 22.4% were classified as simple battery. Consistent positive associations were found between extreme heat and DV-related calls; for example, the likelihood of a DV-related call increased by 4% (odds ratio, 1.04; 95% CI, 1.02-1.07) when the mean UTCI was above 30 °C. Stronger associations were seen during prolonged exposure to extreme heat; when the mean UTCI exceeded the 90th percentile for 5 consecutive days, the likelihood of a DV-related call increased by 7% (odds ratio, 1.07; 95% CI, 1.03-1.12). Eliminating such heat wave events would most likely prevent approximately 245.0 DV-related calls (95% CI, 105.1-370.9) during the study period.

**Conclusions and Relevance:**

In this cross-sectional study of the association between extreme heat and DV-related calls in New Orleans, DV-related calls were associated with extreme heat events, with the strongest associations observed during prolonged heat waves. These findings highlight the need to integrate climate adaptation with violence prevention strategies during such extreme heat events.

## Key Points

**Question:**

Is extreme heat exposure associated with increased domestic violence–related calls to the New Orleans Police Department?

**Findings:**

In this cross-sectional study of 150 523 domestic violence–related calls from 2011 to 2021 aggregated at the zip code day level, extreme heat events were significantly associated with increased domestic violence calls. The strongest association was observed for heat waves defined using percentile-based thresholds that lasted for 5 or more consecutive days.

**Meaning:**

This study suggests that climate adaptation strategies should be integrated with violence prevention efforts during extreme heat events to reduce the risk of domestic violence.

## Teaser

This cross-sectional study examines whether heat exposure was associated with increased domestic violence–related calls for service in New Orleans, Louisiana, from 2011 to 2021.

## Introduction

The association between climate and human behavior has long been a subject of scientific inquiry, with extensive literature documenting how ambient temperature can influence both the incidence and severity of violent behavior. Research has identified multiple pathways through which higher temperatures may increase interpersonal violence, particularly domestic violence (DV). These include a biological pathway wherein high temperatures lead to reduced self-control and aggression by influencing serotonin neurotransmission in the brain.^1,2^ Other theories include the temperature-aggression hypothesis^3,4^ and the general affective aggression model^5^ in psychology, both of which support the notion of uncomfortable hot temperatures as a mechanism directly inducing aggressive motivation and behavior.

Although this mechanism may help explain the general association between heat and aggression, it is particularly important to understand how it manifests in domestic settings where power dynamics and gender-based vulnerabilities create additional risk factors. There is little research examining the potential association of extreme heat events with DV, particularly in the US.^6^ The few existing studies, largely from outside the US, show that heat waves precede incidents of intimate partner violence (IPV), as indicated by self-reported experiences, emergency calls, and femicide data.^1,7,8,9^ This gap in knowledge is concerning for cities such as New Orleans, Louisiana, which is characterized as one of the most climate-vulnerable urban areas in the US.^10^ The city’s vulnerability is compounded by high rates of DV; approximately one-third of Louisiana residents report a lifetime experience of physical or sexual IPV, with women more likely to experience such violence.^11^ Louisiana has consistently ranked among the top 10 states for female homicide in more than half of the past 25 years,^12^ with New Orleans recently experiencing a 12-year peak in DV-related 911 calls in May 2023.^13^ Given these concerns and the projected increase in heat wave intensity and frequency, it is important to understand the association between extreme heat and DV in the context of New Orleans. Such an understanding can inform violence prevention efforts.

This study examines the association between extreme heat events and subsequent DV-related calls to the New Orleans Police Department (NOPD) between 2011 and 2021. We hypothesize that the risk of such calls would be higher under extreme heat conditions.

## Methods

### DV-Related Calls Data

The records of calls for service made to the NOPD between January 1, 2011, and December 31, 2021, were provided by the Orleans Parish Communication District.^14^ Data collection ended in 2021 because, from 2022, the city adjusted its classification of calls related to DV.^15^ The information available in the publicly available data included the date of the call, the zip code and block address from where the call was made, and the type of call made. We classified all calls as DV related or “other” based on the type of call data as recommended by the Domestic Violence Annual Report released by the City of New Orleans.^16^ The following call types were classified as DV related: domestic aggravated battery, simple battery domestic, aggravated assault domestic, simple assault domestic, attempted homicide domestic, domestic criminal damage, simple burglary domestic, domestic threats, and disturbance domestic. The Tulane University institutional review board deemed the study exempt from review as the data used were deidentified and publicly available. The cross-sectional study followed the Strengthening the Reporting of Observational Studies in Epidemiology (STROBE) reporting guideline. All the data used are publicly available.

We limited our analysis to DV-related calls (N = 150 532). Of these, 827 had missing information on the zip codes. For these cases, we used the block address to obtain the zip code using Google Maps and were able to identify the zip codes for 818 cases, resulting in 150 523 cases with known zip code and call dates. We aggregated this dataset to zip code days, which led to 52 447 zip code days. A few zip codes had very few calls during the entire study period (n ≤ 36), and we excluded these from the analysis, resulting in a final sample of 52 399 zip code days of DV-related calls.

### Extreme Heat Data

We obtained daily gridded Universal Thermal Climate Index (UTCI) data, derived from ERA5 reanalysis (European Centre for Medium-Range Weather Forecasts Reanalysis, version 5).^17^ This dataset had a spatial resolution of 0.25° × 0.25° and ranged from January 1, 1970, to December 31, 2021. Using the UTCI instead of air temperature allowed us to account for the effects of wind speed, radiation, humidity, and air temperature and, therefore, better capture the human physiological reaction to multidimensional thermal conditions.^18^ Using long-term daily data enabled us to establish location-specific baseline climate distributions at each zip code, which were essential for calculating percentile-based thresholds for extreme heat events. This approach created definitions of extreme heat that reflected truly anomalous conditions relative to the local climate, following established methods in climate epidemiology research,^19^ and has been extensively used to study the health effects of extreme temperatures.^20^

We used area-weighted averaging to aggregate the 0.25° gridded UTCI data to zip code boundaries. Specifically, for each zip code, we calculated the weighted mean UTCI value where the weight for each grid cell was determined by the proportion of that cell’s area intersecting with the zip code polygon. This ensured that partially overlapping grid cells contributed proportionally to the final zip code–level UTCI value based on their spatial overlap.

### Exposure Variables

For each day of our study period, we constructed 6 definitions of extreme heat using different thresholds for the mean UTCI and the number of consecutive days when its values exceeded the respective threshold. First, we considered an absolute threshold of 30 °C and identified extreme heat days when the mean UTCI was above this threshold. We then constructed heat wave definitions when the mean UTCI was greater than this threshold for 3 and 5 consecutive days leading up to the case-day. In addition, we used a percentile-based threshold, using the 90th percentile of the long-term distribution of the mean UTCI at the zip code of residence, calculated over a 6-day window around the day of interest, from January 1, 1970, up to the case date.

### Statistical Analysis

Statistical analysis was conducted from March to May 2024. We used a spatially weighted, time-stratified case-crossover design to assess the association between extreme heat and DV-related calls in New Orleans.^21^ We identified case days as days when NOPD reported 1 or more DV-related calls. We matched these days with control days, identified as days within the same day of week, month, and year specific to the zip code. The case-crossover design controlled for time-invariant confounders by design.^22^ Although adjusting for time-varying confounders could theoretically strengthen the model, identifying the true confounders in temperature-health associations is challenging. Most relevant time-varying variables (eg, seasonal patterns, day of week effects, and long-term trends) are already captured through time stratification. Variables that vary with temperature, such as air pollutants, are typically either not true confounders or represent intermediate variables on the causal pathway rather than confounders.^23,24^ We, therefore, did not consider these variables as confounders.

Next, we fit a conditional logistic regression model and weighted the model by the number of cases reported on each zip code day to consider the spatial variability in the incident cases. We calculated the attributable fraction and attributable number using odds ratios (ORs) from the conditional logistic regression model. We derived the attributable fraction using the formula (OR − 1)/OR. We used a Monte Carlo simulation approach with 1000 iterations to generate empirical confidence intervals (eCIs), and we used the 2.5th and 97.5th percentiles of the resulting distribution to form the lower and upper confidence bounds.^25,26^ We computed the attributable number by multiplying the attributable fraction by the total number of DV-related calls during exposed days, which was calculated as the product of exposed days and mean daily calls during exposure. We generated the eCIs for the attributable number through the same Monte Carlo process.

To assess the robustness of our findings, we conducted several sensitivity analyses considering alternative definitions of extreme heat exposure. We examined the associations using different UTCI (28 °C, 30 °C, and 32 °C) and percentile-based (85th, 90th, and 95th) thresholds. For each threshold, we considered duration by heat exposures ranging from a single day to 5 consecutive days above the threshold. This allowed us to evaluate the sensitivity of the association between extreme heat and DV-related calls to how extreme heat was operationalized. All statistical analysis was conducted using R, version 4.4.1 (R Project for Statistical Computing).

## Results

### Types of Calls

Of the total 150 523 DV-related calls made to the NOPD during the study period, 69.6% were classified as domestic disturbance, and 22.4% were classified as simple battery (Table 1). The remaining 8.0% of calls comprised criminal damage domestic, aggravated assault domestic, extortion (threats) domestic, aggravated battery domestic, simple assault domestic, simple burglary domestic, and homicide domestic.

**Table 1.** Distribution of Types of Domestic Violence–Related Calls Made to the New Orleans Police Department Between 2011 and 2021

Type of call	No. (%) (N = 150 523)
Domestic disturbance	104 704 (69.6)
Simple battery domestic	33 719 (22.4)
Criminal damage domestic	4825 (3.2)
Aggravated assault domestic	2468 (1.6)
Extortion (threats) domestic	2346 (1.6)
Aggravated battery domestic	1203 (0.8)
Simple assault domestic	1036 (0.7)
Simple burglary domestic	205 (0.1)
Homicide domestic	17 (0.01)

### Association Between Extreme Heat and DV-Related Calls

**Figure 1.**
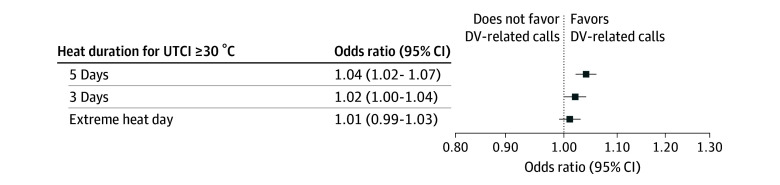
Associations Between Extreme Heat and Domestic Violence (DV)–Related Calls in New Orleans, 2011-2021 Separate conditional logistic regression models were constructed for each exposure. Exposures correspond to a Universal Thermal Climate Index (UTCI) of 30 °C or more on the day the call was made (extreme heat day) and for days when the UTCI was above the threshold for 3 days (heat wave: 3 days) and 5 days (heat wave 5 days).

**Figure 2.**
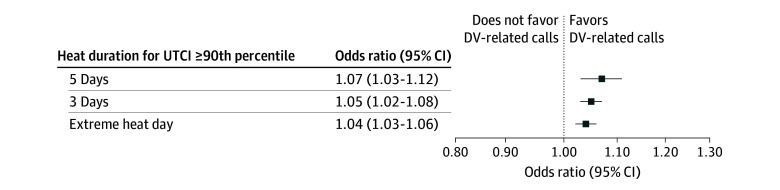
Associations Between Extreme Heat and Domestic Violence (DV)–Related Calls, New Orleans, 2011-2021 Separate conditional logistic regression models were constructed for each exposure. Exposures correspond to a Universal Thermal Climate Index (UTCI) greater than or equal to the 90th percentile of the historic distribution specific to zip code on the day the call was made (extreme heat day) and for days when the UTCI was above the threshold for 3 days (heat wave: 3 days) and 5 days (heat wave 5 days).

We found a positive association between extreme heat and DV-related calls in New Orleans for both absolute and percentile-based thresholds, especially for heat waves lasting for 5 or more consecutive days. For example, the likelihood of DV-related calls increased by 4% (OR, 1.04; 95% CI, 1.02-1.07) when the mean UTCI was above 30 °C for 5 or more consecutive days (Figure 1). A slightly stronger association was observed when the mean UTCI exceeded the 90th percentile cutoff for 5 or more consecutive days. For such extreme heat events, we observed an increased likelihood of 7% for DV-related calls (OR, 1.07; 95% CI, 1.03-1.12) (Figure 2).

Sensitivity analyses revealed distinct patterns for absolute and percentile-based temperature thresholds. For absolute thresholds, we found minimal associations when using a 28 °C UTCI as the threshold (eTable 1 in Supplement 1), but stronger associations emerged at higher temperatures. Although, in our main analysis, we observed modest increases in DV-related calls during 3-day heat waves (OR, 1.02; 95% CI, 1.00-1.04) using a 30 °C UTCI as the threshold (Figure 1), the associations were amplified when using a 32 °C UTCI as the threshold (OR, 1.08; 95% CI, 1.04-1.13) (eTable 1 in Supplement 1). For percentile-based thresholds, we observed a clearer dose-response association across different cutoff points. Associations were generally stronger and estimates more precise compared with absolute thresholds. Starting from a single extreme heat day using the 85th percentile threshold (OR, 1.04; 95% CI, 1.02-1.05) (eTable 1 in Supplement 1), associations gradually strengthened with higher thresholds and heat wave durations. The strongest associations were observed using the 95th percentile threshold, with 5-day heat waves showing the most pronounced association (OR, 1.15; 95% CI, 1.08-1.23), although these estimates had wider 95% CIs due to fewer exposure days.

### Attributable Fraction of DV Cases

Our results also demonstrate a general pattern that increased duration of heat exposure was associated with an increase in the attributable fraction of DV cases as well. This pattern was particularly pronounced for heat events defined using the 90th percentile threshold. For example, when examining prolonged heat exposure (defined as an UTCI ≥90th percentile threshold for 5 consecutive days), we found that eliminating such heat wave events would reduce DV cases by approximately 7% (attributable fraction, 7.0; 95% eCI, 3.0-10.5), corresponding to 245.0 fewer DV-related calls during our study period (95% eCI, 105.1-370.9) (Table 2).

**Table 2.** Attributable Fraction and Attributable Number of Domestic Violence Cases by Heat Exposure Definition

Heat exposure definition	Attributable fraction (95% empirical CI)	Attributable No. (95% empirical CI)
**Universal Thermal Climate Index ≥30 °C**		
Extreme heat day	1.2 (−1.0 to 3.3)	306.9 (−259.7 to 849.5)
Heat wave: 3 d	1.9 (−0.3 to 4.1)	341.5 (−53.3 to 729.9)
Heat wave: 5 d	4.1 (1.8 to 6.6)	532.6 (235.0 to 868.2)
**Universal Thermal Climate Index ≥90th percentile**		
Extreme heat day	4.3 (2.6 to 5.9)	978.0 (580.5 to 1333.8)
Heat wave: 3 d	4.8 (2.3 to 7.4)	379.1 (179.3 to 588.6)
Heat wave: 5 d	7.0 (3.0 to 10.5)	245.0 (105.1 to 370.9)

Our estimates of attributable fractions and attributable numbers from sensitivity analyses generally support the main results, with some variations across different thresholds. The absolute temperature–based thresholds (28 °C and 32 °C) showed inconsistent results, with some producing negative or nonsignificant estimates, particularly for longer heat wave durations (eTable 2 in Supplement 1). In contrast, the percentile-based definitions (85th and 95th percentiles) demonstrated consistent dose-response patterns across all exposure durations (eTable 3 in Supplement 1). The 95th percentile threshold revealed the strongest association for 5-day heat waves (attributable fraction, 13.2; 95% eCI, 7.2-18.4), suggesting that the most extreme heat events have been disproportionately associated with DV. These findings reinforce that percentile-based heat definitions provide more robust estimates than absolute temperature thresholds of the association between extreme heat and DV.

## Discussion

Our findings contribute to the growing body of literature on climate risks and violence against women,^6^ and they demonstrate an association between heat exposure and DV-related calls in New Orleans, based on more than a decade of data. Our results are aligned with other studies that also found a heat-violence connection. An interdisciplinary meta-analysis by Hsiang et al^27^ found each SD increase in temperature to be associated with a 4% increase in interpersonal violence. A study across 57 countries concluded similarly, reporting a 6% increase in homicide with every temperature increase of 1 °C.^28^ Studies specifically looking at the association between heat and IPV also had similar findings. Sanz-Barbero et al^7^ studied this phenomenon in Madrid, Spain, and found an increase in intimate partner femicides, police reports of IPV, and use of an IPV telephone help line after a heat wave.^7^ Zhu et al^8^ used survey data from India, Pakistan, and Nepal and found every 1 °C increase in the annual mean temperature was associated with a mean increase in IPV prevalence of approximately 5%.

Our results show 2 important patterns. First, the associations are more pronounced when using the 90th percentile threshold compared with the absolute 30 °C UTCI threshold, and second, the association between heat and DV-related calls indicates an increasing trend of DV-related calls with increasing duration of heat exposure. Our sensitivity analyses using multiple temperature thresholds and exposure definitions largely corroborated our main findings, demonstrating the robustness of the observed association between extreme heat and DV-related calls. These findings taken together suggest that prolonged exposure to heat may have cumulative associations with interpersonal stress and aggressive behavior. Although both absolute and percentile-based thresholds showed positive associations, the more consistent estimates observed with percentile-based thresholds (especially at higher thresholds) suggest that relative temperature measures may be more appropriate for studying heat-behavior associations in this context. This aligns with increasing evidence that local adaptation and acclimatization play important roles in how communities respond to extreme heat and underscores the need to consider local temperature patterns when developing climate adaptation strategies.

### Strengths and Limitations

This study has some strengths. It provides evidence of the association between extreme heat and DV-related calls, using more than a decade of data and using multiple analytical approaches that consistently demonstrated this association. The use of both absolute and relative temperature thresholds, along with various exposure durations, strengthens our confidence in these findings. Furthermore, our case-crossover design effectively controlled for time-invariant confounders, while our zip code–specific analyses helped account for local variations in heat exposure and response patterns.

Several important limitations must be considered when interpreting these results. First, our reliance on emergency calls data likely underestimates the true burden of DV, as many incidents go unreported. This underreporting bias could be more pronounced during extreme heat events if individuals experiencing DV face additional barriers to seeking help. The direction of this bias would likely lead to conservative estimates of the heat-violence association. Regarding external validity, New Orleans presents a unique context, with its subtropical climate, historical patterns of racial and ethnic segregation, and specific socioeconomic challenges. The city’s vulnerability to extreme weather events and limited adaptive capacity may make our findings less generalizable to cities with a more robust infrastructure or different climatic conditions. However, the biological and behavioral mechanisms linking heat stress with aggressive behavior are likely consistent across populations, suggesting that our findings may be relevant to other urban areas experiencing extreme heat events.

Finally, the available data limited our geographic resolution to the zip code level, which may mask important neighborhood-level variations in both heat exposure and DV patterns. This aggregation could obscure significant sociospatial disparities, particularly in a city such as New Orleans, where historical patterns of segregation and disinvestment have led to marked differences in environmental conditions and social vulnerability between neighborhoods, even within the same zip code.

Future research can address these limitations by incorporating multiple data sources beyond emergency calls, such as hospital records, DV shelter data, and police reports, to better capture the full spectrum of DV incidents. Studies could also benefit from more granular geographic data and the inclusion of neighborhood-level characteristics, such as tree canopy cover, building density, and socioeconomic indicators. In addition, research examining the interaction between extreme heat and other environmental stressors (such as air pollution or flooding) could provide a more comprehensive understanding of how climate change is associated with DV. Finally, comparative studies across different cities with varying climatic conditions and adaptive capacities could help establish the generalizability of these findings and inform climate adaptation strategies that incorporate DV prevention. Such research could provide useful guidance to support urban investments and policies to support heat mitigation infrastructures.^29^

## Conclusions

In this cross-sectional study of the association of extreme heat with DV-related calls in New Orleans, we found that DV-related calls were positively associated with extreme heat events, with the strongest associations observed during prolonged heat waves (defined using percentile-based thresholds) lasting 5 or more consecutive days, where the likelihood of DV-related calls increased between 6% and 15% (corresponding to the 85th and 95th percentile thresholds), and elimination of such extreme heat events prevented a significant number of DV cases over the study period.

This study highlights critical public health concerns, identifying extreme heat as a potential risk factor for IPV. The findings emphasize the need for targeted interventions and messaging as well as resource allocation during extreme heat events, especially among vulnerable urban populations. In addition, this study highlights 911 emergency calls as a critical source of data on DV prevalence in the city. Greater care in categorization of such calls could further refine the city’s efforts to combat crime and improve health outcomes as they relate to violence against women.^30^ The study’s findings also call for integrating climate change adaptation with violence prevention strategies and public education efforts, creating a more holistic approach to addressing these intersecting issues.

## Notes

Supplement 1. **eTable 1.** Odds Ratios and 95% Confidence Intervals for the Association Between all Exposure Definitions and Domestic Violence Calls Made in New Orleans Between 2011 and 2021 **eTable 2.** Attributable Fraction and Attributable Number of Domestic Violence Cases by Absolute Temperature-Based Heat Exposure Definitions **eTable 3.** Attributable Fraction and Attributable Number of Domestic Violence Cases by Percentile-Based Heat Exposure Definitions

Supplement 2.
Data Sharing Statement
