# Supplementary material for: Extreme Heat and Calls to Law Enforcement Related to Domestic Violence
Source: JAMA Netw Open. 2025 Aug 29;8(8):e2530530. doi: 10.1001/jamanetworkopen.2025.30530 (PMC12397886; doi:10.1001/jamanetworkopen.2025.30530)
Supplement: Supplement 1. — eTable 1. Odds Ratios and 95% Confidence Intervals for the Association Between all Exposure Definitions and Domestic Violence Calls Made in New Orleans Between 2011 and 2021 eTable 2. Attributable Fraction and Attributable Number of Domestic Violence Cases by Absolute Temperature-Based Heat Exposure Definitions eTable 3. Attributable Fraction and Attributable Number of Domestic Violence Cases by Percentile-Based Heat Exposure Definitions [file jamanetwopen-e2530530-s001.pdf]

Supplementary Online Content

Dey AK, Rao N, Thomas EE, et al. Extreme heat and calls to law enforcement related to domestic violence. *JAMA Netw Open.* 2025;8(8):e2530530. doi:10.1001/jamanetworkopen.2025.30530

eTable 1. Odds Ratios and 95% Confidence Intervals for the Association Between all Exposure Definitions and Domestic Violence Calls Made in New Orleans Between 2011 and 2021
eTable 2. Attributable Fraction and Attributable Number of Domestic Violence Cases by Absolute Temperature-Based Heat Exposure Definitions
eTable 3. Attributable Fraction and Attributable Number of Domestic Violence Cases by Percentile-Based Heat Exposure Definitions

This supplementary material has been provided by the authors to give readers additional information about their work.

© 2025 Dey AK et al. *JAMA Network Open.*

eTable 1. Odds Ratios and 95% Confidence Intervals for the Association Between all Exposure Definitions and Domestic Violence Calls Made in New Orleans Between 2011 and 2021

Heat Exposure Durations	Odds Ratio (95% Confidence Interval)	Odds Ratio (95% Confidence Interval)
	**UTCI >= 28°C**	**UTCI >= 85th percentile**
Extreme heat day	1.03 (1.01, 1.06)	1.04 (1.02, 1.05)
Heatwave: 2 days	1.02 (0.99, 1.04)	1.04 (1.02, 1.05)
Heatwave: 3 days	1.00 (0.98, 1.03)	1.05 (1.02, 1.07)
Heatwave: 4 days	0.99 (0.97, 1.01)	1.03 (1.01, 1.06)
Heatwave: 5 days	0.99 (0.97, 1.01)	1.06 (1.02, 1.09)
	**UTCI >= 30°C**	**UTCI >= 90th percentile**
Extreme heat day	1.01 (0.99, 1.03)	1.04 (1.03, 1.06)
Heatwave: 2 days	1.01 (0.99, 1.03)	1.06 (1.03, 1.08)
Heatwave: 3 days	1.02 (1.00, 1.04)	1.05 (1.02, 1.08)
Heatwave: 4 days	1.02 (1, 1.05)	1.05 (1.01, 1.08)
Heatwave: 5 days	1.04 (1.02, 1.07)	1.07 (1.03, 1.12)
	**UTCI >= 32°C**	**UTCI >= 95th percentile**
Extreme heat day	1.02 (0.99, 1.05)	1.05 (1.02, 1.07)
Heatwave: 2 days	1.05 (1.02, 1.09)	1.03 (1, 1.06)
Heatwave: 3 days	1.08 (1.04, 1.13)	1.02 (0.99, 1.07)
Heatwave: 4 days	1.08 (1.03, 1.14)	1.04 (0.99, 1.09)
Heatwave: 5 days	1.04 (0.98, 1.1)	1.15 (1.08, 1.23)

© 2025 Dey AK et al. *JAMA Network Open.*

eTable 2. Attributable Fraction and Attributable Number of Domestic Violence Cases by Absolute Temperature-Based Heat Exposure Definitions

Heat Exposures	Attributable Fraction (95% Empirical Confidence Interval)	Attributable Number (95% Empirical Confidence Interval)
**UTCI >= 28°C**		
Extreme heat day	3.15 (1.06, 5.25)	1338.15 (449.33, 2230.55)
Heatwave: 2 days	1.48 (-0.71, 3.65)	568.93 (-270.63, 1400.47)
Heatwave: 3 days	0.41 (-1.95, 2.52)	143.76 (-685.69, 885.41)
Heatwave: 4 days	-0.63 (-3.01, 1.47)	-203.68 (-971.58, 473.33)
Heatwave: 5 days	-1.02 (-3.01, 1.17)	-303.4 (-894.36, 346.59)
**UTCI >= 30°C**		
Extreme heat day	1.17 (-0.99, 3.25)	306.87 (-259.7, 849.51)
Heatwave: 2 days	0.84 (-1.09, 2.82)	178.44 (-231.22, 595.3)
Heatwave: 3 days	1.91 (-0.3, 4.07)	341.53 (-53.28, 729.86)
Heatwave: 4 days	2.08 (-0.43, 4.44)	318.49 (-65.05, 679.29)
Heatwave: 5 days	4.07 (1.8, 6.63)	532.59 (234.96, 868.23)
**UTCI >= 32°C**		
Extreme heat day	1.88 (-1.32, 4.73)	139.02 (-97.4, 349.91)
Heatwave: 2 days	5.15 (1.6, 8.36)	255.74 (79.37, 415.62)
Heatwave: 3 days	7.8 (3.97, 11.55)	267.65 (136.12, 396.41)
Heatwave: 4 days	7.73 (2.94, 12.52)	173.41 (65.91, 280.87)
Heatwave: 5 days	3.57 (-2.9, 9.49)	50.24 (-40.78, 133.36)

© 2025 Dey AK et al. *JAMA Network Open.*

eTable 3. Attributable Fraction and Attributable Number of Domestic Violence Cases by Percentile-Based Heat Exposure Definitions

**Heat Exposures**	**Attributable Fraction (95% Empirical Confidence Interval)**	**Attributable Number (95% Empirical Confidence Interval)**
**UTCI >= 85th percentile**		
Extreme heat day	3.73 (2.38, 5.13)	1178.83 (750.94, 1620.92)
Heatwave: 2 days	3.48 (1.72, 5.14)	693.23 (342.41, 1023.59)
Heatwave: 3 days	4.37 (2.44, 6.49)	558.92 (311.45, 829.72)
Heatwave: 4 days	3.2 (0.59, 5.77)	268.68 (49.26, 485.16)
Heatwave: 5 days	5.28 (2.35, 8.24)	308.72 (137.53, 482.06)
**UTCI >= 90th percentile**		
Extreme heat day	4.3 (2.55, 5.86)	978.04 (580.49, 1333.81)
Heatwave: 2 days	5.25 (3.23, 7.31)	684.82 (421.93, 953)
Heatwave: 3 days	4.79 (2.27, 7.44)	379.05 (179.28, 588.63)
Heatwave: 4 days	4.41 (1.13, 7.53)	224.46 (57.41, 383.21)
Heatwave: 5 days	6.96 (2.98, 10.53)	245.00 (105.06, 370.90)
**UTCI >= 95th percentile**		
Extreme heat day	4.32 (2.47, 6.42)	547.85 (313.78, 814.96)
Heatwave: 2 days	3.08 (0.33, 5.65)	204.69 (21.8, 375.41)
Heatwave: 3 days	2.43 (-1.92, 6.17)	83.65 (-66.26, 212.72)
Heatwave: 4 days	3.76 (-1.19, 8.43)	73.58 (-23.23, 164.99)
Heatwave: 5 days	13.24 (7.21, 18.41)	168.93 (92.02, 234.95)

© 2025 Dey AK et al. *JAMA Network Open.*
