# Supplementary material for: Extreme Heat and Calls to Law Enforcement Related to Domestic Violence
Source: JAMA Netw Open. 2025 Aug 29;8(8):e2530530. doi: 10.1001/jamanetworkopen.2025.30530 (PMC12397886; doi:10.1001/jamanetworkopen.2025.30530)
Supplement: Supplement 2. — Data Sharing Statement [file jamanetwopen-e2530530-s002.pdf]

Data Sharing Statement

Dey. Extreme Heat and Calls to Law Enforcement Related to Domestic Violence. *JAMA Netw Open*. Published August 29, 2025. doi:10.1001/jamanetworkopen.2025.30530

Data
Data available: Yes
Data types: Data (not involving human participants)
How to access data: https://github.com/benmarhnia-lab/heat-DV-calls-NOLA
When available: beginning date: 02-15-2025

Supporting Documents
Document types: Statistical/analytic code
How to access documents: https://github.com/benmarhnia-lab/heat-DV-calls-NOLA
When available: beginning date: 02-15-2025

Additional Information
Who can access the data: The data and corresponding analytical code are available on a public GitHub repository
Types of analyses: For research
Mechanisms of data availability: With investigator support
